# The Fabry disease-associated lipid Lyso-Gb3 enhances voltage-gated calcium currents in sensory neurons and causes pain

**DOI:** 10.1016/j.neulet.2015.01.084

**Published:** 2015-05-06

**Authors:** L. Choi, J. Vernon, O. Kopach, M.S. Minett, K. Mills, P.T. Clayton, T. Meert, J.N. Wood

**Affiliations:** a Molecular Nociception Group, Wolfson Institute for Biomedical Research, University College London, Gower St., London, WC1E 6BT, UK; b BK21 Programme, Department of Molecular Medicine, Seoul National University, South Korea

**Keywords:** Gb3, globotriaosylceramide, Lyso-Gb3, lyso-globotriaosylceramide, GLA, gene encoding alpha galactosidase A, HBSS, HEPES buffered saline, PBS, phosphate buffered saline, Pain, Calcium imaging, Voltage-dependent Ca^2+^ channels, Fabry disease, Dorsal root ganglia

## Abstract

Fabry disease is an X-linked lysosomal storage disorder characterised by accumulation of glycosphingolipids, and accompanied by clinical manifestations, such as cardiac disorders, renal failure, pain and peripheral neuropathy. Globotriaosylsphingosine (lyso-Gb3), a deacylated form of globotriaosylceramide (Gb3), has emerged as a marker of Fabry disease. We investigated the link between Gb3, lyso-Gb3 and pain. Plantar administration of lyso-Gb3 or Gb3 caused mechanical allodynia in healthy mice. *In vitro* application of 100 nM lyso-Gb3 caused uptake of extracellular calcium in 10% of sensory neurons expressing nociceptor markers, rising to 40% of neurons at 1 μM, a concentration that may occur in Fabry disease patients. Peak current densities of voltage-dependent Ca^2+^ channels were substantially enhanced by application of 1 μM lyso-Gb3. These studies suggest a direct role for lyso-Gb3 in the sensitisation of peripheral nociceptive neurons that may provide an opportunity for therapeutic intervention in the treatment of Fabry disease-associated pain.

## Highlights

• Gb3 and Lyso-Gb3, plasma lipids accumulating in Fabry disease, cause mechanical allodynia in mice.
• Lyso-Gb3 elevates intracellular calcium level in sensory neurons.
• Lyso-Gb3 enhances voltage-dependent calcium currents in small-diameter DRG neurons.
• Direct effects of lyso-Gb3 on sensory neurons may contribute to the pain of Fabry disease.

## 1 Introduction

Fabry disease (OMIM 301500) is a lysosomal storage disorder caused by a deficiency or absence of the enzyme α-galactosidase A, due to mutations of the X-linked gene *GLA* [1]. As a result, unmetabolised glycosphingolipids accumulate in various types of cells including neurons of the dorsal root ganglia (DRG) [2]. Accumulation in the lysosomes of vascular endothelial cells contributes to a characteristic renal and cardiovascular pathology. Neuropathic pain, typically a sensation of burning, itching or shooting pain in the hands and feet, is an early symptom of Fabry disease [2–4]. However, little is known about the direct effects of accumulated lipids on neuronal function.

Among the glycosphingolipids which are found to be elevated in patients with Fabry disease, globotriaosylceramide (Gb3) has been recognised as a diagnostic and predictive marker [5]. However, recent studies suggest the existence of other factors in addition to Gb3, since GLA enzyme replacement therapy for clearance of excessive Gb3 does not result in remission, and Gb3 levels do not always correlate with severity [6]. Globotriaosylsphingosine (lyso-Gb3), a deacylated form of Gb3, has emerged as a good biomarker for Fabry disease since a robust increase in plasma in both patients and animal models has been reported [7]. Lyso-Gb3 has been proposed to play a causative role in Fabry disease pathogenesis by accelerating Gb3 storage [7]. In GLA null mutant mice and in Fabry patients, the drug migalastat-HCl lowers plasma levels of lyso-Gb3, which suggests that the lyso marker is clinically relevant [8,9], although phase III drug trial endpoints were not met [10]. Migalastat both mimics the terminal galactose on Gb3, and stabilises the structure of mutant GLA, while it is targeted to the lysosome where Gb3 can be metabolised. However, the contribution of lyso-Gb3 to Fabry pain is uncertain.

Pain is a response to noxious stimuli, such as cold or heat, mechanical stress or the signals arising during inflammation. The noxious stimulus is detected by peripheral damage-sensing neurons (nociceptors). The depolarisation of the nociceptor is usually linked to an increase in cytosolic Ca^2+^ levels [19]. Although accumulation of lipids in DRG has been reported in Fabry patients [2], the effect of elevated glycosphingolipids on sensory neuron function has not been investigated. We therefore, examined whether lyso-Gb3 functionally affects nociceptive neurons to determine if there is a causal link between the marker lyso-Gb3 and pain in Fabry disease.

## 2 Material and methods

### 2.1 Animals

All experiments were approved by the UCL ethics board with prior approval by the UK home office. C57/BL6 mice (Charles River, UK) were treated in accordance with the UK Animal (Scientific Procedures) Act 1986.

### 2.2 Von Frey test

Mechanical withdrawal thresholds were measured by applying von Frey hairs (Stoelting, Wood Dale, USA) to the plantar surface of the hindpaw. The 50% paw withdrawal threshold was calculated using the up-and-down method [11]. Animals received a plantar injection of Gb3, lyso-Gb3 or saline (20 μl) 30 min prior to testing. All experiments were performed blind to treatment.

### 2.3 Adult DRG neuronal culture

DRG from all spinal levels were dissected from adult 7–8 week old mice and cultured as previously described [12]. Dissociated cells were plated on 1 μg/ml poly-l-lysine and 0.02 mg/ml laminin pre-coated either 15 mm cover slips (for calcium imaging) or 35 mm petri dishes (for whole-cell patch clamp recording).

### 2.4 Calcium imaging

Plated DRG cells were washed with Ca^2+^ recording buffer consisting of (in mM) 140 NaCl_2_, 4 KCl, 2CaCl_2_, 1 MgCl_2_, 10 HEPES, 10 glucose adjusted to pH 7.4 and osmolality 320 mOsm/l using NaOH and sucrose, respectively. Cells were incubated in recording buffer with 2.5 μM Fura-2AM (Invitrogen, UK) for 30 min at room temperature. In some experiments, cells were pre-treated with 5 μg/l IB4-FITC (Sigma, UK) in culture medium at 37 °C for 20 min before Fura-2AM loading. Cells were washed twice with Ca^2+^ recording buffer for 5 min each time at room temperature to remove excess Fura-2AM. A coverslip of cells was placed over the window of a SA-NIK stage adapter (Warner Instruments). The perfusing chamber was tightened onto the adapter, with vacuum grease silicone (Beckman, USA) to enhance the seal. 0.5 ml of Ca^2+^ recording buffer was loaded onto the perfusing chamber before the whole assembly was mounted onto the stage of a Nikon Eclipse TE300 microscope. The inlet tube of a perfusing system (cFlow 8 Channel Flow Controller, Cell Micro Controls, USA) was connected to the recording chamber, and the speed of superfusate was set to 250 μl/min. Images were obtained using either a 20× or 40× (oil immersion) Nikon objective, equipped with fluorescent filter sets. A Till polychrome II monochromator was used to excite the fluorescence of Fura-2 at 340 nm and 380 nm, and data were acquired using OptoFluor software (Cairn Research, UK). Fura-2AM emission was collected at 505 nm.

### 2.5 Electrophysiology

Whole-cell patch-clamp recordings were performed at room temperature using an Axopatch 200B amplifier and Digidata board 1320A controlled by pClamp v9.0 software (Axon Instruments, Molecular Devices Inc.). Recording pipettes were pulled from borosilicate glass microcapillaries (Intracel Ltd., Herts, UK) with a range of pipette resistances between 2 and 4 MΩ after filling with intracellular solution consisting of (in mM) 146 CsCl_2_, 1 CaCl_2_, 4 MgCl_2_, 10 HEPES–Na, 10 EGTA, 4.5 MgATP and 0.4 GTP–Na, adjusted to pH 7.3 and osmolarity 300 mOsm/l using CsOH and sucrose, respectively. Recordings were performed in voltage-clamp mode and currents were low-pass filtered at 5 kHz and sampled at 10 kHz. Values for series resistance (Rs) and capacitance (Cm) were read directly from the amplifier after subtraction of capacitative transients. Series resistances were compensated to the maximum extent possible (usually 60–75%) and voltages were not corrected for liquid junction potentials. External solution consisting of (in mM) 162.5 tetraethylammonium (TEA)–Cl, 5 CaCl_2_, 1 MgCl_2_, 10 glucose and 10 HEPES with pH adjusted to 7.4 using TEA–OH was used to isolate calcium currents. Lyso-Gb3 was applied to cells *via* a perfusing system and the speed of perfusing solution was set at 1 ml/min. Only small-diameter DRG neurons (with capacitance less than 25 pF) were chosen for recording. Off-line data analysis was performed using ClampFit v9.0 software (Axon Instruments, Molecular Devices Inc.).

*Drugs* stocks of Fura-2AM (Invitrogen, UK) and lyso-Gb3 (Sigma, UK) were dissolved in DMSO and HBSS containing 2 mM Ca^2+^ and 1 mM Mg^2+^, respectively. Stocks of capsaicin (8-methyl-*N*-vanillyl-*trans*-6-nonenamide, Sigma, UK) were prepared in ethanol. For intraplantar administration, Gb3 or lyso-Gb3 (Matreya LLC, UK) were prepared in saline. 50 mM KCl was prepared in Ca^2+^ recording buffer on the same day for use.

### 2.6 Statistical analysis

One way ANOVA was followed by a Bonferroni *post-hoc* test when statistical significance was observed. Comparison between two groups was performed with independent samples *T*-test, and a Student’s paired *T*-test was used to compare two different conditions in the same cell unless otherwise stated. Data were presented as the mean ± SEM. All statistical analyses were performed using Prism 5 for Windows.

## 3 Results

### 3.1 Treatment with Gb3 or lyso-Gb3 induces mechanical allodynia in healthy mice

**Fig. 1:**
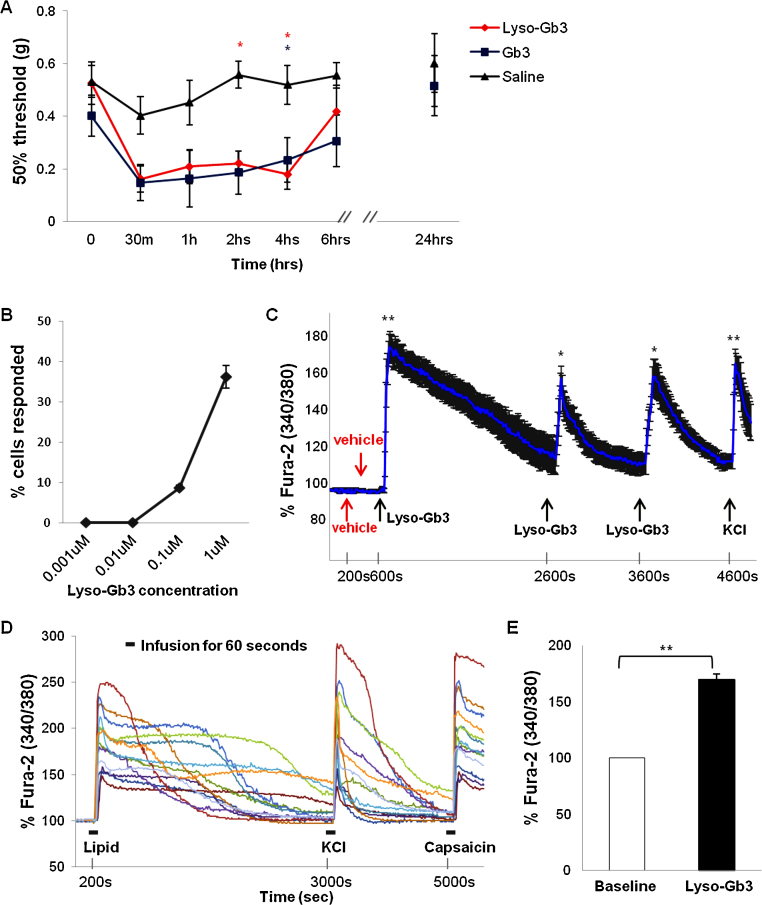
Effects of lyso-Gb3 on pain sensitivity and intracellular Ca^2+^ levels in nociceptive DRG neurons. (A) Either Gb3 or lyso-Gb3 injected into mice hind paws significantly enhanced pain sensitivity to mechanical stimuli over several hours post injection (*n* = 6–9 per group). Sensitivity resolved to a normal level within 24 h. Repeated measures ANOVA revealed effects of time: *F* (6,126) = 7.551, *P* < 0.0001, and of Fabry lipids (*F* (21,126) = 3.089, *P* < 0.001) but no difference between the two lipids (effect of lipid type: *F* (2,126) = 6.179, *P *< 0.05). (B) Lyso-Gb3 evoked an increase in cytoplasmic Ca^2+^ levels in DRG neurons in a dose-dependent manner. The concentration of lyso-Gb3 producing changes in Ca^2+^ levels is plotted against the percentage of responsive DRG neurons. (C) Repetitive applications of lyso-Gb3 (1 μM), but not vehicle evoked transient increases in Ca^2+^ levels in DRG neurons. (D) Representative real time recordings of the changes in Fura-2 ratio acquired every 10 s in individual DRG neurons during bath applications of 1 μM Lyso-Gb3, 50 mM KCl and 1 μM capsaicin. (E) Statistical summary of the changes in Fura-2 ratio before and after application of 1 μM of lyso-Gb3 (*n* = 74). *P < 0.05 and **P < 0.0001.

We administered 20 μl of 30 μM Gb3 or lyso-Gb3 to the hind paws of wild type mice and tested sensitivity at different time points. As seen in Fig. 1A, both Fabry lipids significantly reduced pain thresholds compared to saline (*P* < 0.05). The increased sensitivity lasted up to 6 h. All the mice completely recovered from the acute mechanical allodynia 24 h post injection.

### 3.2 Exogenous lyso-Gb3 increases intracellular Ca^2+^ in sensory neurons

We next examined the cellular actions of lyso-Gb3 on DRG sensory neurons in culture. We investigated the effect of applying lyso-Gb3 on the levels of intracellular Ca^2+^ of DRG neurons. Increased Ca^2+^ levels were observed following lyso-Gb3 application at concentrations higher than 0.1 μM (Fig 1B). Concentrations between 0.1 and 1 μM are found in Fabry disease patients with clinical manifestations [13,14]. Our results indicate that clinical lyso-Gb3 concentrations cause a concentration-dependent increase in Ca^2+^ levels in the cytoplasm of sensory neurons. We then used 1 μM lyso-Gb3 to test if repetitive application of lipid could trigger a repetitive rise in intracellular Ca^2+^ level in DRG neurons. Fig. 1C demonstrates that whilst applications of vehicle alone did not affect basal Ca^2+^ levels, 1 μM lyso-Gb3 applied three times evoked transient increases in Ca^2+^ levels. Administration of high-potassium-containing medium (KCl, 50 mM) evoked a depolarization-mediated rise in intracellular Ca^2+^ level in every tested neuron (Fig. 1C and D). All DRG neurons showing an increase in Ca^2+^ levels following lyso-Gb3 application and responding to 50 mM of KCl were capsaicin-sensitive as shown by an increase in cytoplasmic Ca^2+^ levels during application of 1 μM capsaicin (Fig. 1D).

### 3.3 Lyso-Gb3 increases intracellular Ca^2+^ in capsaicin-sensitive peptidergic neurons

**Fig. 2:**
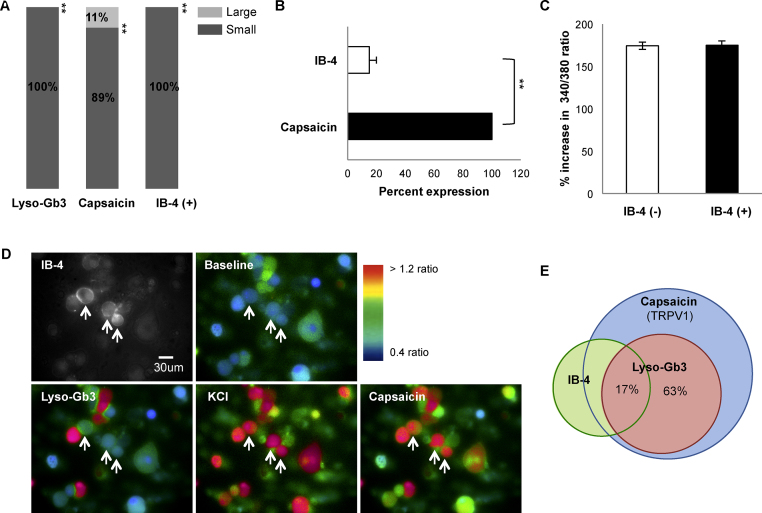
Identification of the DRG neuron population responsive to lyso-Gb3. (A) Distribution of cells according to diameter; lyso-Gb3 responders were all small-diameter cells, as were 89 % of capsaicin-responsive cells. The third bar of the histogram signifies that IB-4 positive cells were all small-diameter neurons. (B) Lyso-Gb3 responsive cells were all stimulated by 1 μM capsaicin, but only 17 % of these bound IB-4.​ (B) Other lyso-Gb3 responsive capsaicin-sensitive cells were IB-4 negative. (C) Statistical summary of the lyso-Gb3-induced changes in Fura-2 ratio in neuronal subgroups shows no significant difference between IB4-positive and IB4-negative DRG neurons. (D) Representative images showing the changes in Fura-2 fluorescence during application of lyso-Gb3, KCl or capsaicin in IB4-positive DRG neurons. Arrows point to the IB4-positive neurons. (E) Subpopulation of lyso-Gb3-responding neurons represents approximately 63% of capsaicin-sensitive DRG neurons, only 17% of those were positive for IB-4 binding. **P < 0.0001.

Sensory neurons can be classified using a range of morphological and functional criteria. Small diameter sensory neurons, many of which are nociceptors, can be subdivided into peptidergic neurons that do not bind the lectin IB4 and another subset that express c-Ret that do bind IB4. [15,16]. These two subsets of sensory neurons have been found to show functional differences [15]. We examined the subgroups of lyso-Gb3 responsive DRG neurons using calcium imaging combined with IB4 labelling. We measured the size of cells which showed at least a 30% increase in Fura-2 ratio following a 1 min superfusion with lyso-Gb3. The lipid responsive cells were identified as small-diameter neurons (Fig. 2A), responsive to 1 μM capsaicin and predominantly negative for IB4 staining – in other words peptidergic neurons (Fig. 2B). However, the average lyso-Gb3-evoked changes in Fura-2 ratio did not differ between IB4-positive and IB4-negative small-diameter DRG neurons (Fig. 2C). Fig. 2E illustrates the overlapping subpopulations detected in these experiments.

**Fig. 3:**
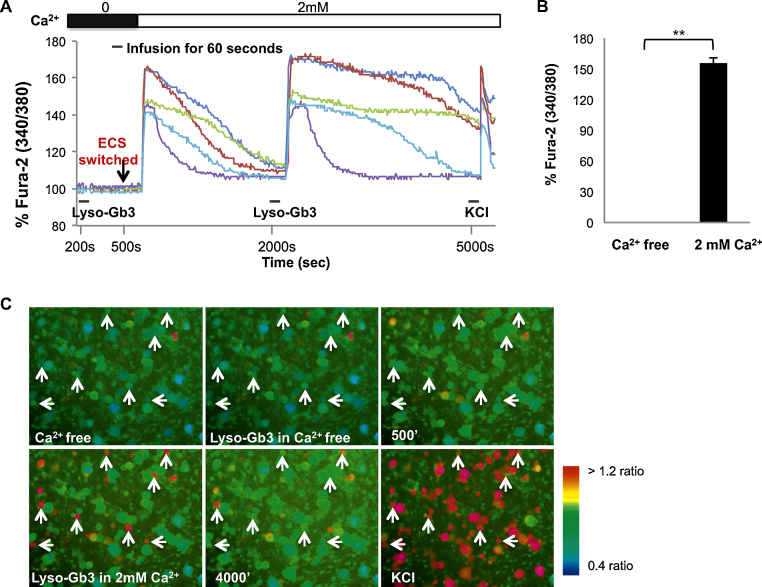
Requirement for extracellular calcium in the Fura-2 response to lyso-GB3. (A) Representative real time traces showing intracellular Ca^2+^ levels following superfusion with lyso-Gb3 in either Ca^2+^ free or standard recording buffer containing 2 mM Ca^2+^. (B) Peak Fura-2AM 340/380 ratio between baseline and lyso-Gb3. Cells appear red in accordance with Ca^2+^ levels. (C) Representative fluorescent images showing changes in Ca^2+^ levels. *Note*: Data sampled every 10 s during calcium imaging. **P < 0.0001.

Fig. 3A shows that increased intracellular Ca^2+^ influx occurred only in the presence of Ca^2+^ in the recording buffer, indicating that the source for increased cytoplasmic Ca^2+^ was the extracellular solution and not intracellular stores.

### 3.4 Lyso-Gb3 increases the current density of voltage-dependent Ca^2+^ channels in small-diameter DRG neurons

**Fig. 4:**
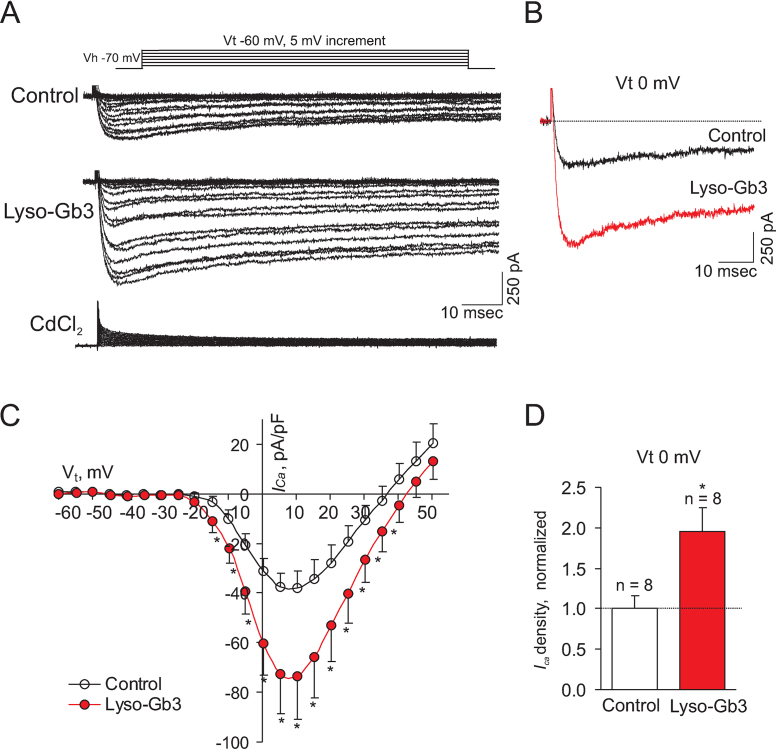
Effects of lyso-Gb3 on Ca^2+^ influx and current density of voltage-activated Ca^2+^ channels in DRG neurons. (A) Representative traces of Ca^2+^ currents evoked in the same small-diameter DRG neuron by voltage steps from holding potential (*V*_h_) –70 mV to test potential (*V*_t_) from –60 mV to +50 mV in 5 mV increments before (top) or during (medium) application of 1 μM lyso-Gb3 *via* a perfusion system. Ca^2+^ currents (bottom) in the presence of lyso-Gb3 were completely inhibited by 50 μM CdCl_2_. Inset illustrates the voltage-clamp protocol used to elicit currents. (B) Representative Ca^2+^ currents recorded at *V*_t_ 0 mV before (control) and after lyso-Gb3 application. (C) Average current–voltage relationships of Ca^2+^ currents before and during an application of 1 μM lyso-Gb3. The peak current amplitudes at each *V*_t_ were normalized to cell capacitance and expressed as the current density. (D) A statistical summary of the current densities at *V*_t_ 0 mV before and during lyso-Gb3 application. Changes in current density were normalized to control.. *P < 0.05 (paired t-test).

Next we examined whether the lyso-Gb3-evoked increases in cytoplasmic Ca^2+^ levels were mediated by effects on voltage-activated Ca^2+^ channels, known to control Ca^2+^ entry during neuronal depolarisation [17]. To assess how lyso-Gb3 affects voltage-activated Ca^2+^ currents in nociceptive DRG neurons, we recorded inward Ca^2+^ currents in small-diameter DRG before (control) and during application of lyso-Gb3 by eliciting currents with a set of 250-ms voltage steps from −60 to +50 mV (*V*_t_) in 5 mV increments (holding potential: –70 mV, see Fig. 4A top). In the presence of 1 μM lyso-Gb3, Ca^2+^ currents were significantly augmented in small-diameter DRG neurons at most of the tested membrane potentials (Fig. 4A and B). Furthermore, the current density of voltage-activated Ca^2+^ currents was significantly increased from *V*_t_ −15 mV to +35 mV (*n* = 8/membrane potential; *P* < 0.05; Fig. 4C and D). In the presence of vehicle for lyso-Gb3 there was no significant change in current before and during the application (data not shown).

## 4 Discussion

This study investigated a potential link between high levels of the Fabry lipid lyso-Gb3 and pain. In healthy mice administration of lyso-Gb3 induced mechanical allodynia. In peptidergic DRG neurons, lyso-Gb3 evoked an increase in intracellular Ca^2+^ levels associated with the functional upregulation of voltage-activated Ca^2+^ channels.

Plasma circulation of lyso-Gb3 and the concomitant lysosomal accumulation of Gb3 have been proposed to lead to pain in Fabry disease [7], and our study provides evidence consistent with this, by showing that healthy mice receiving plantar injections of Gb3 or lyso-Gb3 developed mechanical allodynia. This observation is consistent with direct sensitization of DRG neurons following infiltration of lysoGb3 into plasma. We found that lyso-Gb3 triggers a rise in intracellular Ca^2+^ levels in a subpopulation of DRG neurons that express markers associated with damage sensing. The origin of excessive lysoGb3 is not clearly identified, although drugs that stabilise the GLA enzyme also reduce the levels of lyso-Gb3 in plasma [9] suggesting that lyso-Gb3 is a direct product of normal lipid catabolism rather than the outcome of degradation.

This study is the first to show a direct link between lyso-Gb3, intracellular changes in peripheral sensory neurons and pain. The pain syndrome in Fabry disease was thought to arise from small fibre dysfunction, such as length dependent neuropathy which is typically linked to a loss or decrease of myelinated Aδ fibres and unmyelinated C fibres [4]. However, it is not known whether the exposure to or accumulation of lipids directly causes small nerve fiber damage [18], and our study did not investigate any neuropathic effects of lyso-Gb3. Nevertheless the possibility of small nerve fibre damage by lyso-Gb3 exists, given that promoted Ca^2+^ influx may cause Ca^2+^-dependent excitotoxicity [19]. Our study showed that only high concentrations of lyso-Gb3 equivalent to the level in pre-treated Fabry patients evoked Ca^2+^ rises in small-diameter DRG neurons of the IB4 nonbinding, peptidergic sub-class.

Intracellular Ca^2+^ signalling regulates many cellular functions. In the present study, 1 μM lyso-Gb3, a possible concentration reported in patients with Fabry disease [13], was shown to induce a promoted membrane Ca^2+^ influx. Further, the lipid enhanced voltage-dependent Ca^2+^ current densities, indicating functional upregulation of voltage-dependent Ca^2+^ channels as a plausible mechanism of functional changes in peripheral nociceptors. Voltage-dependent Ca^2+^ channels in DRG neurons play a role in transmission of nociceptive signals from the periphery, and neuropathic manifestations including increased pain sensitivity have also been shown to be associated with increased Ca^2+^ entry in disease states, such as diabetes [20].

This is the first report showing the onset of mechanical allodynia, and augmented Ca^2+^ influx mediated by upregulated voltage-dependent Ca^2+^ channels in nociceptive DRG neurons, following lyso-Gb3 administration. This study shows that Fabry lipids may cause pain through direct actions on sensory neurons, and that promoted Ca^2+^-dependent excitability of nociceptors is a possible mechanism. These observations support further investigation of the effects of Fabry-disease associated lipids on ion fluxes in sensory neurons and their potential significance in the pain associated with this condition.
